# Clinical characteristics and treatment outcomes of tuberculosis in the elderly: a case control study

**DOI:** 10.1186/1471-2334-13-121

**Published:** 2013-03-05

**Authors:** Yong Soo Kwon, Su Young Chi, In Jae Oh, Kyu Sik Kim, Yu Il Kim, Sung Chul Lim, Young Chul Kim

**Affiliations:** 1 Department of Internal Medicine, Chonnam National University Hospital, Gwangju, South Korea

**Keywords:** Age, Clinical presentations, Diagnosis, Treatment outcome, Tuberculosis

## Abstract

**Background:**

The purpose of this study was to evaluate the differences in clinical characteristics and treatment outcomes between older and younger tuberculosis (TB) patients in Korea.

**Methods:**

We retrospectively analyzed the medical records of 271 younger (20–64 years old at diagnosis) and 199 older (≥65 years) TB patients who had been newly diagnosed and treated at Chonnam National University Hospital from May 2008 to August 2010.

**Results:**

Dyspnea and comorbid medical conditions were more frequent and positive TB culture rates were higher in older TB patients. In chest computed tomography (CT) scans of pulmonary TB patients, older patients were less likely to have micronodules (<7 mm in diameter), nodules (<30 mm in diameter), masses (>30 mm in diameter), and cavities compared with younger patients, but were more likely to have consolidations. Incidence of adverse drug reactions did not differ between the two groups, except for severe gastrointestinal disorders. There were no significant differences in favorable treatment outcomes between younger and older TB patients (97% vs. 94%, respectively; p = 0.251).

**Conclusions:**

Older TB patients had more frequent dyspnea and less frequent active TB findings on chest CT. Treatment success and adverse drug reaction rates were similar in older and younger TB patients.

## Background

The increase in the number of elderly people due to improved life expectancy presents special challenges to the control of tuberculosis (TB) in South Korea. In this country, the notification rate of active TB remains as high as 98.4 per 100,000 general population and 266.0 per 100,000 in adults aged ≥65 years [1].

It has been suggested that TB presentation in older patients may differ from that in younger patients and should be classified as a separate entity [2,3]. These individuals have more comorbidity, increasing the risk of active TB and altering its presenting symptoms [4]. Although several studies have described the differences between older and younger TB patients, there were some discordant findings on clinical presentation, radiological findings, laboratory features, and treatment outcomes [2-5]. Chest computed tomography (CT) is superior to chest radiography in the diagnosis of pulmonary TB [6-9]. However, there are no data about differences in chest CT findings between younger and older TB patients.

In an effort to clarify this issue, here we describe the differences between older and younger TB patients concerning their presenting symptoms, diagnostic findings, especially focused on chest CT findings, treatment outcomes, and adverse drug reactions.

## Methods

### Study population

We enrolled all adult patients aged >20 years who were treated for newly diagnosed active TB at the Chonnam National University Hospital between May 2008 and August 2010. We retrospectively collected data for clinical, radiographic, and bacteriological status of these patients. From 542 patients with newly diagnosed active TB, we excluded those who displayed non-tuberculous mycobacteria in final culture results (n = 15), and those who had been transferred to another institution after being treated for <3 months at our hospital (n = 57). This left 470 patients with newly diagnosed active TB who received anti-TB medication for >3 months. Permission was obtained from the Institutional Review Board of Chonnam National University Hospital to review and publish patient records retrospectively. Informed consent was waived because of the retrospective nature of the study.

Active TB comprised two situations. The first was definite cases that were confirmed by a positive culture or nucleic acid amplification test for *Mycobacterium tuberculosis* in clinical specimens. The second was probable cases with caseous granuloma in tissue histology results or typical findings of active TB on radiological examinations, high clinical suspicion, positive tuberculin skin test or interferon γ release assay (IGRA), and good clinical responses to anti-TB treatment without a culture-positive result or nucleic acid amplification test for *M. tuberculosis*.

Elderly patients were defined as persons aged ≥65 years at the time of TB diagnosis. They were compared with younger patients (20–64 years old at diagnosis). In South Korea, a 6-month self administered regimen consisting of a 2-month initial phase of isoniazid, rifampicin, pyrazinamide, and ethambutol, followed by a 4-month continuation phase of isoniazid, rifampicin, and ethambutol has been recommended by the National Tuberculosis Program [10]. Alternatively, a 9-month regimen with isoniazid, rifampin, and ethambutol can be administered [10]. Therefore, most patients in this study received daily therapy consisting of isoniazid (300 mg), rifampin (450–600 mg), ethambutol (0–1200 mg), and pyrazinamide (1500 mg). However, the decision to include pyrazinamide in the initial regimen was made by clinicians based on each patient’s clinical situation.

### Microbiological and radiological evaluations

In patients with suspected pulmonary TB, sputum acid fast bacilli (AFBs) stain and mycobacterial cultures were performed more than three times according to the recommendation of the Korean TB guidelines [11]. In extrapulmonary TB, AFB stain, mycobacterial cultures, and TB polymerase chain reaction (PCR) were performed if there were available patient samples. Sputum TB PCR was routinely performed on the first sample for testing of AFB stains. We performed bronchoscopy if patients could not expectorate sputum or had negative AFB smear results for their sputa and had chest images that could not differentiate from other diseases such as a lung malignancy, fungal infections, and parasitic infestations. All specimens were decontaminated and cultured on liquid media using the BACTEC MGIT 960 system (Becton Dickinson, Sparks, MD, USA). *M. tuberculosis* was identified by multiplex PCR assay as described previously [12]. For all multiplex PCRs, amplification was performed in a GeneAmp PCR 9600 DNA thermal cycler (Perkin–Elmer, Waltham, MA, USA). IGRA was performed using a QuantiFERON-TB Gold In-Tube (Cellestis, Carnegie, Vic, Australia) and the results were interpreted as specified by the manufacturer.

Chest CT scans were performed in all patients suspected of having active pulmonary TB and could not be differentiated from other diseases such as lung cancer or pneumonia at the start of treatment. We evaluated chest CT findings for evaluation of the lesions including [9,13]: (1) micronodule: a nodule with a diameter <7 mm; (2) nodule: a focal, round opacity with a diameter <3 cm; (3) mass: a round opacity with a diameter >3 cm; (4) tree-in-bud sign: nodular dilatation of centrilobular branching structures that resembled a budding tree; (5) consolidation: a homogeneous increase in lung opacity that resulted in obscuration of underlying vessels; (6) ground glass opacity: a hazy increase in lung opacity that was not associated with obscuration of underlying vessels; and (7) bronchiectasis: bronchial dilatation, often with thickening of the wall. In addition, the presence of cavitation, fibrotic changes, lymphadenopathy, and pleural thickening with effusion were noted.

### Treatment outcomes and adverse drug reactions

For this analysis, we applied the definitions in the World Health Organization recommendations concerning cure, treatment completed, treatment failure, death, default, and transfer out [14]. For purposes of analysis, cure or treatment completed were assigned to favorable outcomes and death, default, and transfer out to unfavorable outcomes.

We evaluated adverse drug reactions that resulted in an interruption and change of treatment with review of medical records including: hepatotoxicity; skin rash or itching sense; gastrointestinal (GI) problems including nausea, vomiting, diarrhea, and dyspepsia; optic neuropathy; and thrombocytopenia.

### Statistical analyses

Values are expressed as the mean ± standard deviation, or as numbers (percentages) in the text and tables. The categorical comparisons of older versus younger adults were assessed using the χ^2^ test. Differences with regard to numeric values between the older and younger patients were analyzed using the Student’s *t* test for variables with a normal distribution. Logistic regression analysis was used to adjust the effects of sex difference, the presence of underlying diseases, and smoking status on clinical presentation between older and younger TB patients. A p value <0.05 was considered to be statistically significant. Null hypotheses of no difference were rejected for p < 0.05, or, equivalently, if the 95% confidence interval (CI) of odds ratio (OR) estimates excluded 1. Calculations were done using SPSS for Windows version 17.0 (SPSS, Chicago, IL, USA).

## Results

### Patient characteristics

Table 1 summarizes the baseline characteristics of the 199 older and 271 younger patients. Older TB patients had a higher prevalence of women than the younger TB patients had. There were more definite cases in the older TB patients than in the younger TB patients (81% vs. 68%, p = 0.002). Among the respiratory symptoms in patients with pulmonary TB, dyspnea was more frequently found in older TB patients compared to younger TB patients. In the definite cases, hemoptysis was more frequently found in younger TB patients than in older TB patients (22% vs. 10%, p = 0.007). However after adjusting for sex, smoking, and comorbid conditions such as cardiovascular disease, diabetes mellitus, and chronic obstructive pulmonary disease (COPD), there were no differences in cough (OR, 1.49; 95% CI, 0.93–2.38), sputum (OR, 1.38; 95% CI, 0.89–2.17), dyspnea (OR, 1.66; 95% CI, 0.93–2.97), and hemoptysis (OR, 0.61; 95% CI, 0.33–1.15) between the two groups. Prevalence of comorbid conditions including cardiovascular diseases, diabetes mellitus, and COPD was higher in older TB patients.

**Table 1:** Baseline patient’s characteristics in older and younger tuberculosis patients

**Characteristics**	**Older (n = 199)**	**Younger (n = 271)**	**p-value**
Gender, male	100 (51)	169 (62)	0.011
Age, median (IQR)	72 (68–76)	44 (32–55)	
BMI, kg/m^2^	21.75 ± 3.59	21.96 ± 3.22	0.420
Ever smoking	87 (44)	129 (48)	0.454
Respiratory symptoms in pulmonary TB*	n = 173	n = 222	
Cough	118 (68)	131 (59)	0.076
Sputum	98 (57)	106 (48)	0.098
Dyspnea	39 (23)	30 (14)	0.027
Hemoptysis	20 (12)	41 (19)	0.081
Weight loss	5 (3)	23 (9)	0.785
General weakness	13 (7)	22 (8)	0.661
Fever	24 (12)	38 (14)	0.644
Night sweating	0 (0)	5 (2)	0.144
History of previous TB	31 (16)	32 (12)	0.275
Comorbid conditions**			
Cardiovascular disease†	75 (38)	24 (9)	<0.001
Diabetes mellitus	43 (22)	32 (12)	0.006
COPD	20 (10)	4 (2)	<0.001
Chronic kidney disease	1 (1)	3 (1)	0.848
Chronic liver disease‡	10 (5)	15 (6)	0.982
Malignancy	9 (5)	9 (3)	0.661

The data are presented mean ± standard deviation for age and pack-years of smoking and as n (%) for all other factors.

*BMI* = body mass index, *COPD* = chronic obstructive pulmonary disease, *IQR* = interquartile range, *TB* = tuberculosis.

*: Pulmonary tuberculosis and pulmonary and extrapulmonary tuberculosis were included.

**: If there were more than one comorbid condition for one patient, all comorbid conditions were recorded.

†: Hypertension and cerebrovascular disease are included.

‡: Hepatitis and liver cirrhosis are included.

### Sites of disease

Table 2 summarizes disease sites of TB in older and younger patients. There were significant differences between the groups according to sites of infection. Pulmonary TB was more frequent in older patients and extrapulmonary TB was more frequent in younger patients. In cases of pulmonary TB, there was no significant difference in lobar predominance (upper lobe vs. middle or lower lobes) between the age groups. However, older TB patients displayed a higher proportion of endobronchial TB (23/173, 13%) than did younger TB patients (12/222, 5%) (p = 0.007). In the case of extrapulmonary TB, older patients had a lower incidence of tuberculous lymphadenitis than younger TB patients had.

**Table 2:** Disease sites in older and younger tuberculosis patients

**Characteristics**	**Older**	**Younger**	**p-value**
Sites of disease	n = 199	n = 271	
Pulmonary TB	155 (78)	183 (68)	0.044
Pulmonary and extrapulmonary TB	18 (9)	39 (14)	
Extrapulmonary TB	26 (13)	49 (18)	
Locations of pulmonary TB	n = 173	n = 222	
Upper*	109 (63)	159 (72)	0.066
Middle or lower	64 (37)	65 (28)	
Locations of extrapulmonary TB†	n = 44	n = 88	
Pleural TB	24 (53)	36 (41)	0.144
Tuberculous lymphadenitis	8 (18)	31 (35)	0.046
Skeletal TB	7 (16)	10 (11)	0.582
Tuberculous pericarditis	4 (9)	2 (2)	0.095
Miliary TB	1(2)	4 (5)	0.664
Tuberculous peritonitis	2 (4)	2 (2)	0.600
CNS TB	0 (0)	1 (1)	1.000
Intestinal TB	0 (0)	3 (3)	0.550
Genitourinary TB	0 (0)	3 (3)	0.550

The data are presented as n (%).

*: Lesion of the upper lobe only or upper lobe with other lobe.

†: Including multiple locations of TB.

*TB* = tuberculosis.

### Diagnostic tests, drug resistance, and radiological findings

The positive rate of TB culture was significantly higher in older than in younger TB patients. Bronchoscopy was more frequently performed in older than in younger TB patients (Table 3). There were no significant differences in isoniazid-resistant TB and multidrug-resistant TB between the two age groups (Table 3).

**Table 3:** Diagnostic tests and drug resistances in older and younger tuberculosis patients

**Characteristics**	**Older**	**Younger**	**p-value**
Positive AFB smear	67/190 (35)	66/247 (27)	0.060
Positive culture for M.TB	155/191 (81)	169/248 (68)	0.002
Positive TB PCR	146/190 (77)	168/247 (68)	0.053
Positive IGRA	56/63 (89)	87/97 (90)	1.000
Positive tuberculin skin test	86/92 (93)	133/143 (93)	1.000
Confirmed by bronchoscopy in pulmonary TB*	64/173 (37)	53/222 (24)	0.005
Drug resistances			
Resistance to INH	10/137 (7)	15/160 (9)	0.665
Multi-drug resistance	1/137 (1)	6/160 (4)	0.185

The data are presented as n (%).

*: included both of the pulmonary tuberculosis, and pulmonary and extrapulmonary tuberculosis.

*IGRA* = interferon gamma releasing assay, *INH* = isoniazid, *MDR* = multi-drug resistant, *M.TB* = *Mycobacterium tuberculosis*, *RFP* = rifampin.

Chest CT findings of pulmonary TB revealed that micronodules, nodules, tree-in-bud appearances, and consolidations were the most common characteristics in pulmonary TB. However micronodules, nodules, masses, tree-in-bud appearance, and cavitations were more frequently found in younger TB patients. Consolidations were more frequently found in older TB patients (Table 4).

**Table 4:** Chest computed tomography findings of pulmonary tuberculosis in older and younger patients

**Findings**	**Older (n = 173)**	**Younger (n = 222)**	**p value**
Micronodules (< 7 mm)	132 (76)	191 (86)	0.018
Nodules (< 30 mm)	60 (35)	159 (72)	<0.001
Masses (> 30 mm)	9 (5)	41 (18)	<0.001
Tree-in-bud appearances	70 (40)	126 (57)	0.016
Consolidations	115 (66)	113 (51)	0.002
Ground glass opacities	34 (20)	30 (14)	0.130
Cavitations	27 (16)	76 (34)	<0.001
Bronchiectasis	28 (16)	39 (18)	0.787
Fibrotic changes	46 (27)	77 (35)	0.100
Lymphadenopathis (>10 mm)	42 (24)	60 (27)	0.564

The data are presented as n (%).

If there are more than one chest computed tomography findings for one patient, all chest computed tomography findings were recorded.

### Treatment outcomes and adverse drug reactions

Of all patients with pulmonary TB, favorable treatment outcomes were achieved in 97% of older patients and 94% of younger patients, with no significant difference (p = 0.251). Concerning unfavorable treatment outcomes between older and younger pulmonary TB patients, there were no significant differences in rates of mortality (2/222 [1%] in younger TB vs. 1/172 [1%] in older TB, p = 1.000), default (10/222 [5%] in younger TB vs. 5/172 [3%] in older TB, p = 0.443), and treatment failure (2/222 [1%] in younger TB vs. 0/172 [0%] in older TB, p = 0.507) (Table 5).

**Table 5:** Treatment outcomes and adverse drug reactions in older and younger tuberculosis patients

**Characteristics**	**Older**	**Younger**	**p-value**
Treatment outcomes in pulmonary TB	n = 173	n = 222	
Favorable	167 (97)	208 (94)	0.251
Unfavorable	6 (3)	14 (6)	
Adverse drug reactions in all patients	n = 199	n = 271	
Hepatotocixity*	6 (3)	13 (5)	0.338
Skin rash or itching	15 (8)	25 (9)	0.642
Gastrointestinal† problems	20 (10)	10 (4)	0.009
Optic neuropathy	2 (1)	8 (3)	0.265
Thrombocytopenia	2 (1)	2 (1)	1.0

The data are presented as n (%).

If there are more than one side effects for one patients, all side effects were recorded.

*: Hepatotoxicity was defined when liver transaminase levels exceeded 120 IU/L.

†:Gastrointestinal problems were problems of gastrointestinal tract for example nausea, vomiting, diarrhea, and dyspepsia etc.

Twenty-one percent of older TB patients and 24% of younger TB patients experienced several adverse drug reactions that resulted in an interruption and change of treatment during anti-TB medication. The adverse events included hepatotoxicity, GI problems, skin rash or itching, optic neuropathy, and thrombocytopenia. Severe GI trouble occurred more frequently in older TB patients, whereas hepatotoxicity occurred more frequently in younger TB patients (Table 5).

## Discussion

In this study, 97% of older TB patients achieved favorable treatment outcome without increased adverse drug reactions, with the exception of GI disorders. They were more likely to have dyspnea and positive culture rates for *M. tuberculosis* compared to younger TB patients. However, active pulmonary TB indicators on chest CT, such as nodules, masses, and cavities, were less frequently found in older TB patients, except for consolidations.

It was previously believed that the treatment outcomes of older TB patients were worse than those of younger TB patients, mainly because of the high mortality rates [15,16]. The mortality rates of older patients have been reported at up to 51% [16,17]. Although these mortality rates have been decreasing recently, the rate remains high. The mortality rate in older TB patients was higher than that in younger TB patients in the United States from 1993 to 2008 (21% vs. 7%, p < 0.001) [16]. Also, in a study conducted in Taiwan, the mortality rate in older pulmonary TB was higher than that in younger patients (27% vs. 4%, p = 0.001) [15]. In the present study, there was no difference in mortality between younger and older TB patients during treatment and the mortality rate was only 0.5% (1/199) in older patients. This was lower than the average mortality of older TB patients in Korea (12%, 1642/14247) in 2010 [1], which could be explained as follows. We excluded patients who received anti-TB therapy for <3 months. However, previous studies and the epidemiological data in Korea have included all TB patients. Therefore, patients who died early in treatment could be excluded from the present study. Another possible cause of the low mortality rate in older TB patients was the high rate of bronchoscopy. Early TB treatment can decrease TB-related mortality; therefore, we believe that the low mortality and high treatment success rates of older patients in our study could reflect the ability of fiberoptic bronchoscopy to achieve a rapid and definitive diagnosis, coupled with the opportunity to test for drug susceptibility. We performed bronchoscopy in 32% (64/199) of older TB patients; this rate was significantly higher than in younger patients (20%, 53/271; p = 0.003). However in our study, the treatment outcomes were measured only at the end of treatment. Therefore, long-term outcomes in TB patients might differ from these results.

Korzeniewska et al. [18] have evaluated the differences in clinical presentations of 218 cases of pulmonary and pleural TB between younger and older patients. Younger patients were more likely to present with fever and night sweats. In culture-confirmed cases, hemoptysis, fever, and cough were more common in younger patients. Chan et al. [19] have also evaluated the differences in 172 bacteriologically (AFB stain and/or culture) or histologically confirmed TB between younger and older patients. Older TB patients had less hemoptysis but more nonspecific symptoms than younger TB patients had. In our study, there were no significant differences in symptoms, except for dyspnea, which was more frequent in older TB patients. This difference could be a result of more prevalent comorbid conditions in older TB patients, because dyspnea did not differ significantly between the groups after adjusting for comorbidity. In respiratory symptoms of pulmonary TB, no consistent differences have been reported between older and younger TB patients in a meta-analysis [4], although in some studies, TB in older patients may present atypically with nonspecific symptoms, which can delay diagnosis [2,3,5,20,21]. These nonspecific symptoms could be the result of comorbid conditions in older TB patients. In a meta-analysis, cardiovascular disorders, COPD, and diabetes were more prevalent in older TB patients [4]. The higher prevalence of hemoptysis in definite cases of younger TB patients in our study, similar to previous studies [4,18], could have been related to the higher frequency of cavities in this population.

Positive tuberculin skin test was also higher in younger TB patients than in older TB patients in a previous study (86% vs. 68%, p = 0.03) [18]. A decrease in immunological status associated with aging could have caused the lower positive rate [4]. However in our study, the positive rate in tuberculin skin test did not differ between the groups. We could not explain the exact cause of the high positive rates in the tuberculin skin test in older TB patients. However, it might be explained by the high level of previous bacille Calmette–Guérin (BCG) vaccination, which in Korea, is given at birth and again at age 12 or 13 years if the child has a negative tuberculin skin test, and presence of latent TB infection, which is as high as 30% among Koreans [22]. However, we do not know the exact number of BCG vaccinations and latent TB infections in the present study.

The prompt diagnosis of active TB is critical for its control program. Imaging could provide rapid diagnosis and early treatment for active TB. However, the radiological patterns of pulmonary TB in older patients have been suggested as atypical, which may differ from those in younger TB patients [23]. Although there was a greater incidence of cavities in younger TB patients and lower lung field lesions in older TB patients compared with the other age group in previous studies, they only compared chest radiographic findings [4,15,24]. The superiority of the chest CT to radiography in evaluating many chest diseases has been established, and many chest CT features of pulmonary TB have been described [6-9]. To the best of our knowledge, the present study is the first recent evaluation of the chest CT pattern in pulmonary TB in a large group of older TB patients. In chest CT findings of pulmonary TB, micronodules, which are not seen on chest radiography, are known as acute inflammatory lesions and are the most common lesions in pulmonary TB [9]. Therefore, these lesions are a useful diagnostic sign of active pulmonary TB. Another important sign of active pulmonary TB on chest CT is a tree-in-bud appearance. This represents a form of bronchiolar impaction in which branching linear structures have more than one contiguous branching site [6,25]. Cavities are the most important sign of pulmonary TB activity [26]. Our data show that older adults with pulmonary TB are less likely to have typical radiological patterns of active pulmonary TB such as micronodules, tree-in-bud appearances and cavities than younger adults with pulmonary TB have, and are more likely to have atypical patterns such as consolidations. These could represent detailed patterns of atypical radiological presentation in older patients with pulmonary TB.

Although there was no significant difference in positive sputum smear results between older and younger TB patients, the former had a higher proportion of positive sputum smears in our study. This is contrary to the present radiological findings that older adults with TB were less likely to have evidence of cavitations and more likely to have atypical patterns on chest CT. Patel et al. have reported the importance of flexible bronchoscopy as a diagnostic tool in the evaluation of pulmonary TB in elderly patients [27]. In our study, fiberoptic bronchoscopy was more frequently performed in older TB patients for differential diagnosis. Older TB patients displayed a higher proportion of bacteriologically proven cases and details of drug susceptibility than younger TB patients did. These findings are similar to those of other studies, in which fiberoptic bronchoscopy was reported as an important diagnostic procedure in patients with suspected pulmonary TB whose sputum specimens were negative both in smear and PCR analyses [28,29].

Advanced age has been shown to raise the risk of hepatotoxicity during anti-TB treatment due to age-related physiological changes and comorbid conditions that require multiple drug therapy [30-35]. However, the influence of age remains a controversial issue concerning the risk of hepatotoxicity during anti-TB treatment because aging has not achieved statistical significance in some studies [36,37]. In the present study, there were no differences in serious adverse reactions, including hepatotoxicity, between the two groups, except for severe GI disorders, which was higher in older TB patients. Different regimens such as the absence/presence of pyrazinamide or treatment duration could cause different rates of adverse drug reactions. However, in the present study, there were no differences in regimens that included pyrazinamide (97%, 264/271) in younger TB patients versus 97% (194/199) in older TB patients (p = 1.000), or in the duration of treatment (237 ± 102 vs. 229 ± 93 days, p = 0.373).

## Conclusions

In summary, older TB patients presented more frequent dyspnea, but on chest CT showed less frequent findings of active pulmonary TB, such as nodules, masses, and cavities. However, the treatment outcome and adverse drug reactions, except for GI disorders, were no different from those in younger TB patients.

## Abbreviations

AFB: Acid fast bacilli;BCG: Bacille Calmette-Guérin;COPD: Chronic obstructive pulmonary disease;CT: Computed tomography;GI: Gastrointestinal;IGRA: Interferon gamma release assay;TB: Tuberculosis

## Competing interests

All authors declare that they have no competing interests.

## Authors’ contributions

Guarantor of integrity of entire study, YSK, study concept and design, YSK and SYC; data acquisition, all authors; data analysis and interpretation, all authors; the manuscript drafting or manuscript revision for important intellectual content, all authors; manuscript final version approval, all authors; literature research, YSK and SYC; clinical studies, YSK, SYC, IJO, and KSK; statistical analysis, YSK, YIK, SCL, and YCK; and manuscript editing, YSK.

## Pre-publication history

The pre-publication history for this paper can be accessed here:

http://www.biomedcentral.com/1471-2334/13/121/prepub
